# Structural Design of Nickel Hydroxide for Efficient Urea Electrooxidation

**DOI:** 10.3390/ma17112617

**Published:** 2024-05-29

**Authors:** Yi Zeng, Shouqin Xiang, Shun Lu, Xueqiang Qi

**Affiliations:** 1 School of Chemistry and Chemical Engineering, Chongqing University of Technology, Chongqing 400054, China; 2 Chongqing Institute of Green and Intelligent Technology, Chinese Academy of Sciences, Chongqing 400714, China

**Keywords:** nickel hydroxide, urea oxidation reaction, surface engineering, structural activity relationship

## Abstract

Urea stands as a ubiquitous environmental contaminant. However, not only does urea oxidation reaction technology facilitate energy conversion, but it also significantly contributes to treating wastewater rich in urea. Furthermore, urea electrolysis has a significantly lower theoretical potential (0.37 V) compared to water electrolysis (1.23 V). As an electrochemical reaction, the catalytic efficacy of urea oxidation is largely contingent upon the catalyst employed. Among the plethora of urea oxidation electrocatalysts, nickel-based compounds emerge as the preeminent transition metal due to their cost-effectiveness and heightened activity in urea oxidation. Ni(OH)_2_ is endowed with manifold advantages, including structural versatility, facile synthesis, and stability in alkaline environments. This review delineates the recent advancements in Ni(OH)_2_ catalysts for electrocatalytic urea oxidation reaction, encapsulating pivotal research findings in morphology, dopant incorporation, defect engineering, and heterogeneous architectures. Additionally, we have proposed personal insights into the challenges encountered in the research on nickel hydroxide for urea oxidation, aiming to promote efficient urea conversion and facilitate its practical applications.

## 1. Introduction

Urea, a prevalent environmental pollutant, originates primarily from industrial processes, agricultural fertilizers, and human urine. The degradation by-products of urea, including compounds such as ammonia and nitrates, may pose risks to human health and the environment [1,2]. However, urea has the advantage of being a liquid fuel, offering an ideal energy density (16.9 MJ L^−1^) and remarkable solubility (1079 g L^−1^ at 20 °C). Simultaneously, it addresses the challenges of toxicity and volatility commonly associated with other fuels [3,4]. Urea oxidation reaction (UOR) technology not only facilitates energy conversion but also contributes significantly to the treatment of wastewater rich in urea [5,6,7]. Therefore, conducting research on UOR offers a dual benefit: addressing the issue of urea-containing wastewater while enhancing H_2_ production efficiency. Firstly, in addressing the degradation of urea-containing wastewater, Diao et al. [8] employed a one-step hydrothermal method to fabricate foam nickel-supported NiFe ultra-thin two-dimensional (2D) nanosheet arrays as UOR electrocatalysts. They constructed a dual-electrode electrolyzer with NiFe NSs/NF serving as the anode and utilizing a solution of 1 M KOH and 0.33 M urea as the electrolyte. Test results revealed that following electrolysis for 36 h at a voltage of 1.7 V, the urea concentration decreased by 55.6%, thus substantiating the feasibility of electrocatalytic urea removal from wastewater. Subsequently, regarding energy-efficient H_2_ production, traditional electrolysis of water involves two half-reactions, namely, the oxygen evolution reaction (OER) (Equation (1)) and the hydrogen evolution reaction (HER) (Equation (2)), with the reaction equations as follows:

4OH^−^ → O_2_ + 2H_2_O + 4e^−^ (1)

2H_2_O + 2e^−^ → H_2_ + 2OH^−^ (2)

Among them, the HER involves a 2-electron transfer reaction, while the OER is a 4-electron-proton-coupled reaction, requiring higher energy, resulting in an oxygen evolution overpotential much higher than the theoretical decomposition voltage of water (1.23 V) [9,10,11]. Additionally, considering the resistance of electrode materials and electrolytes, as well as the contact resistance between electrodes and electrolytes, the actual operating voltage is usually above 1.8 V. However, urea electrolysis operates at a notably lower total voltage of 0.37 V under standard conditions [12,13,14,15,16]. This is achieved with the assistance of urea addition, a species more easily oxidized as a substitute for oxygen evolution, enhancing the energy efficiency of hydrogen production through electrolysis (Equations (3)–(5)) [17,18,19,20].

Urea electrolysis for H_2_ generation:

Anode:

(3) $$\mathrm{CO}{\left({\mathrm{NH}}_{2}\right)}_{2}+6OH^{-}\to \;N_{2}\;+5H_{2}O+{\mathrm{CO}}_{2}+6e^{-}$$

Cathode:

(4) $$6H_{2}O+6e^{-}\to \;3H_{2}\;+6OH^{-}$$

Overall reaction:

(5) $$\mathrm{CO}{\left({\mathrm{NH}}_{2}\right)}_{2}+H_{2}O\;\to \;N_{2}\;+3H_{2}+{\mathrm{CO}}_{2}$$

Nevertheless, the urea electro-oxidation process, characterized by 6-electron transfer, introduces complexity to the reaction mechanism, contributing to sluggish reaction kinetics [21,22,23]. To bolster the efficiency of urea oxidation, there is a pressing need for the development of effective and stable UOR catalysts [24].

Despite considerable research endeavors aimed at developing efficient electrocatalysts for the UOR, there remains ample space for the continued design and development of UOR electrocatalysts that are both efficient and cost-effective [25,26,27,28]. Among the myriad of UOR electrocatalysts, nickel stands out as the most extensively employed transition metal element in current research, owing to its combination of relatively low cost and high UOR activity [29,30,31].

Single-metal nickel-based catalysts exhibit drawbacks such as inadequate active sites, poor conductivity, and low stability. Consequently, researchers have redirected their focus towards Ni-based sulfides, phosphides, and hydroxides. However, the synthesis procedures for sulfides and phosphides are intricate, and certain sulfides and phosphides pose environmental concerns. Thus, taking into account considerations of synthesis methodologies and environmental implications, researchers shifted their attention towards Ni-based hydroxides. Nickel hydroxide (Ni(OH)_2_) has attracted considerable attention owing to its versatile structure, easy preparation, abundant three-dimensional electron supply, and stability in alkaline mediums [32,33,34,35]. Nickel hydroxide primarily exists in two forms: α-Ni(OH)_2_ and β-Ni(OH)_2_. The β-Ni(OH)_2_ crystalline structure adopts a hexagonal brucite configuration featuring a hexagonally dense arrangement of OH^−^ ions. Nickel atoms strategically occupy the interlayer spaces within an octahedral framework. In contrast, α-Ni(OH)_2_ showcases a distinctive layered spiral architecture composed of evenly spaced NiO_2_ layers [36,37]. This structure incorporates OH⁻ ions and certain metal ions, creating a sophisticated arrangement between adjacent NiO_2_ layers. Boggs et al. proposed that in alkaline media, the electrooxidation of urea on Ni catalysts follows a direct oxidation mechanism, where urea molecules are electrochemically oxidized on the surface of NiOOH catalysts, as elucidated by Equations (6)–(9) [38,39,40].

Anode:

(6) $${\mathrm{Ni}(\mathrm{OH})}_{2}+OH^{-}⇌\mathrm{NiOOH}+H_{2}O\;+\;e^{-}$$

(7) $$\mathrm{CO}{\left({\mathrm{NH}}_{2}\right)}_{2}+6OH^{-}\to \;N_{2}\;+5H_{2}O+{\mathrm{CO}}_{2}+6e^{-}$$

Cathode:

(8) $$5H_{2}O+6e^{-}\to 3H_{2}+6OH^{-}$$

Overall reaction:

(9) $$\mathrm{CO}{\left({\mathrm{NH}}_{2}\right)}_{2}+H_{2}O\;\to \;N_{2}\;+3H_{2}+{\mathrm{CO}}_{2}$$

Expanding on the previously discussed reactions, an in-depth investigation into the UOR catalytic pathway under alkaline conditions was conducted using the density functional theory (DFT) [41]. According to experimental observations, competitive adsorption reactions between OH^−^ ions on the NiOOH surface involve both adsorbed ions and urea molecules. During the urea decomposition process, the step that governs the reaction rate is associated with the release of CO_2_. Prior research has expounded upon the potential mechanisms of UOR facilitated by NiOOH under alkaline conditions, encompassing direct oxidation and an indirect or electrocatalyst regeneration mechanism (EC) [42,43]. Conforming to the mechanism of direct oxidation, the electrochemical oxidation of urea takes place, catalyzed by NiOOH, as depicted by Equations (6) and (7). These findings also suggest the feasibility of urea oxidation through an indirect mechanism or catalyst regeneration (EC) on NiOOH catalysts.

Anode:

(10) $$E\;{6\mathrm{Ni}(\mathrm{OH})}_{2}+6OH^{-}⇌6\mathrm{NiOOH}+6H_{2}O\;+\;{6e}^{-}$$

(11) $$C\;6\mathrm{NiOOH}+\mathrm{CO}{\left({\mathrm{NH}}_{2}\right)}_{2}+H_{2}O\to {6\mathrm{Ni}(\mathrm{OH})}_{2}\;+N_{2}+{\mathrm{CO}}_{2}$$

Net anodic reaction:

(12) $${\mathrm{EC}}_{\;}\mathrm{CO}{\left({\mathrm{NH}}_{2}\right)}_{2}+6OH^{-}\to N_{2}+5H_{2}O+{\mathrm{CO}}_{2}+6e^{-}$$

In an alkaline environment, pursuant to the indirect oxidation mechanism, Ni(OH)_2_ undergoes electrochemical oxidation, yielding NiOOH, serving as the medium that facilitates the process of urea oxidation. Throughout the progression of urea oxidation, the catalytic activity of NiOOH undergoes chemical reduction, leading to the formation of the inert Ni(OH)_2_ [44,45]. Concurrently, urea undergoes chemical oxidation to produce its respective products (Equations (10)–(12)). Owing to the elevated oxidation potential, the inherent inactivity of Ni(OH)_2_ results in the subsequent electrochemical oxidation, reverting to NiOOH for catalyst regeneration. This sequence further facilitates the oxidation of urea molecules. However, NiOOH exhibits a strong affinity for intermediates in the reaction, hindering the cycling between Ni(OH)_2_ and NiOOH and consequently diminishing the oxidation efficiency of UOR. Simultaneously, the poor electronic conductivity of Ni(OH)_2_ and the confinement of active sites within its planar structure pose additional barriers to enhancing its UOR performance. Therefore, engineering approaches are required to address the aforementioned issues and enhance the UOR activity of Ni(OH)_2_.

**Figure 1:**
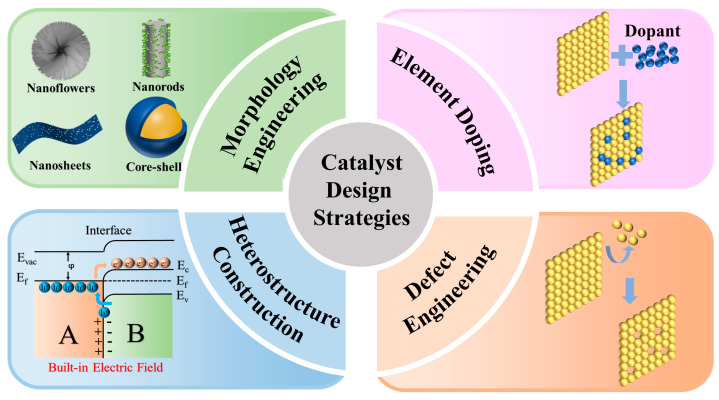
Schematic diagram of Ni(OH)_2_ design strategy.

This study extensively investigates a range of common strategies, including morphology design, heteroatom doping, surface vacancies, and heterogeneous structures (Figure 1), with the goal of enhancing the UOR activity of Ni(OH)_2_. Notably, our previous work related to nickel hydroxide via rational design towards efficient urea electrooxidation is limited [11]. For example, we proposed several strategies, such as morphological design, heteroatom doping, and supporting materials selection for the engineering of nickel hydroxide. Herin, a comprehensive review of the latest research progress in utilizing Ni(OH)_2_ catalysts for UOR is provided, placing particular emphasis on highlighting key examples. Lastly, the central focus was on overcoming obstacles and investigating the possibilities associated with the effective utilization of Ni(OH)_2_ in advancing UOR.

## 2. Catalyst Design Engineering Principles

The design of catalyst morphology stands as a pivotal strategy in modulating catalyst performance. Within the field of UOR electrocatalysts, morphological design is primarily geared towards achieving dual objectives: augmenting the specific surface area and fabricating a porous or 3D network to facilitate unimpeded mass transport. The incorporation of porosity serves a dual function, enhancing both the specific surface area and the establishment of continuous mass transport pathways. For urea electrooxidation, the catalytically active phase resides in Ni^3+^. A larger specific surface area facilitates the exposure of a richer array of active sites, thereby catalyzing the oxidation of a greater portion of Ni^2+^ to Ni^3+^. Furthermore, the continuous pathways formed by porous or networked structures ensure comprehensive electrolyte–catalyst interaction, facilitating the expulsion of reaction by-products like CO_2_. This prevents the occupation of active sites and thus promotes the oxidation of urea. It is worth noting that edge atoms, possessing surplus unoccupied positions adjacent to neighboring atoms, are rendered more accessible to reactants, thereby exhibiting a heightened propensity to participate in catalytic reactions [46,47]. Additionally, the distinctive crystal structure surrounding edge atoms may engender a disparate electron density compared to interior atoms. This heightened electron density surrounding edge atoms bolsters their conductivity, thereby expediting the 6-electron transfer process inherent to urea oxidation. Crafting catalysts into configurations enriched with edges and corners, such as nanosheets or nanorods, and diminishing thickness can amplify the abundance of edge atoms. However, an excess of edge atoms may convolute electron transfer pathways, impeding electron transfer, while excessively thin profiles may compromise the mechanical robustness of the catalyst. Hence, we contend that in morphological design, the paramount objective should revolve around optimizing catalyst performance by concurrently magnifying specific surface area and furnishing continuous mass transport conduits. The emergence of edge atoms should be viewed as an incidental dividend rather than the primary pursuit.

Besides morphology design, element doping is also an important means to modify catalysts. The introduced elements can alter the original crystal and electronic structures of catalysts. Doping atoms typically replace some atoms at certain positions within the crystal lattice. Doping atoms typically substitute specific lattice sites within the crystal structure. Owing to potential disparities in size between the doping atoms and the intrinsic atoms of the crystal lattice, structural adjustments occur to accommodate the presence of the doping atoms. This change enhances the exposure of active sites on the catalyst, generating more edge sites, thereby improving catalytic activity. The altered crystal structure further induces changes in the catalyst’s surface electronic structure. Differences in electronic structure between doping atoms and Ni atoms result in changes in local charge distribution, subsequently affecting electron cloud density and ultimately modifying the catalyst’s surface electronic structure. This modification influences the electron affinity of the catalyst surface, thereby adjusting the energy levels of active sites and facilitating easier adsorption of urea and intermediates onto the catalyst surface, thus enhancing the catalyst’s activity in the urea oxidation process [48].

Defect engineering constitutes a pivotal strategy within catalyst design, embodying a synthesis reminiscent of both morphology design and element doping. Through the deliberate introduction of irregular defects onto the catalyst’s surface, often resulting in the formation of pore or cavity structures, a profound augmentation of the catalyst’s specific surface area ensues. Particularly in the context of urea electrooxidation, this augmentation furnishes an abundance of active sites conducive to the generation of NiOOH. Concurrently, the strategic formation of defects within the catalyst interior ingeniously engenders edge atoms, thereby inducing alterations in electron density at defect sites and fortifying the catalyst’s electrical conductivity. Moreover, the introduction of defects within or upon the material’s structure facilitates the manipulation of lattice or electronic configurations, thereby enhancing the exposure of active sites and fine-tuning the adsorption dynamics of intermediates during urea oxidation. This orchestrated interplay ultimately serves to expedite the urea oxidation process [49]. Notably, in contrast to traditional doping methodologies, defect engineering operates within localized domains, affording a heightened degree of controllability and precision.

One of the strategies for improving catalyst activity is the use of heterogeneous structure materials, which go beyond simple composites. The coupling effect between different materials at the heterogeneous interface surpasses the concept of traditional composite materials, allowing heterogeneous structure materials to exhibit superior mechanical or physical properties during the reaction process while maintaining long-term stability. More importantly, once they come into contact, it leads to the bending of the energy bands of one of the materials due to the difference in the work functions of the materials, thereby promoting the transfer of electrons to the interface until the work functions of the two materials reach equilibrium. This spontaneous electron transfer results in the formation of a built-in electric field at the heterogeneous interface, not only accelerating charge transfer but also enhancing the adsorption capacity for amino and carbonyl groups in urea oxidation reactions, thereby increasing the rate of urea oxidation [50].

## 3. Catalyst Design Strategies

### 3.1. Morphology Engineering

**Figure 2:**
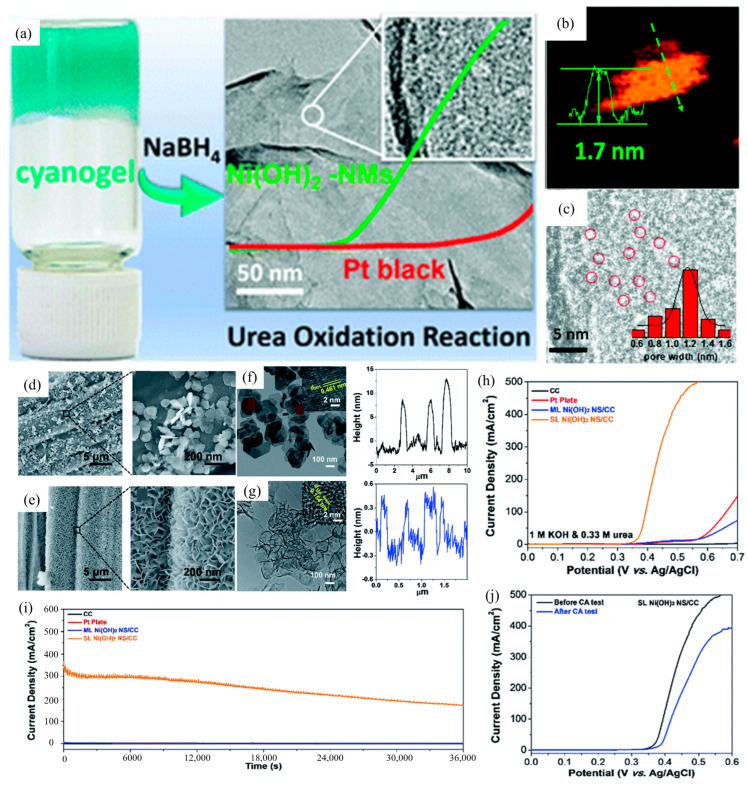
(**a**) The image of the NiCl_2_–K_2_Ni(CN)_4_ cyanogen and (**b**,**c**) AFM and magnified HR-TEM image of Ni(OH)_2_ nano meshes, inset in (**c**): histogram of aperture distribution of the pore size, reprinted with permission from Ref. [51]. Copyright 2019, The Royal Society of Chemistry. (**d**,**e**) SEM of two kinds of Ni(OH)_2_ NS/CC, (**f**,**g**) TEM of ML and SL Ni(OH)_2_ NS/CC and related height profiles, (**h**) LSV curves of different catalysts, (**i**) CA tests for different catalysts, and (**j**) LSV of Ni(OH)_2_ NS/CC before and after the CA test, reprinted with permission from Ref. [52]. Copyright 2018, The Royal Society of Chemistry.

The UOR activity of catalysts is strongly affected by factors such as specific surface area and active site quantity, often addressed through the design of catalyst shapes to optimize material geometry. The nanosheet structure is a typical representative of a high surface area. Ding et al. [51] synthesized Ni(OH)_2_ nanosheets with an atomic thickness of 1.7 nm, atomic force microscopy (AFM) analysis, and high-resolution transmission electron microscopy (HR-TEM) indicated that the acquired Ni(OH)_2_-NMs exhibit a substantial surface area along with multiple nanopores, providing highly active catalytic sites and good mass transfer, demonstrating excellent electrocatalytic UOR activity (Figure 2a–c). Lin et al. [52] employed methanol-guided in situ synthesis to fabricate single-layer α-Ni(OH)_2_ (SL α-Ni(OH)_2)_ on carbon cloth (CC) with a thickness of only ~0.8 nm. Taking advantage of the excellent conductivity of CC (Figure 2d–g), they demonstrated exceptional UOR performance through linear sweep voltammetry, achieving a current density of 436.4 mA cm^−2^ at 0.5 V vs. Ag/AgCl (Figure 2h). Moreover, thanks to the formation of a 3D porous structure, SL α-Ni(OH)_2_ NS/CC has not only excellent UOR activity but also good stability. Compared with ML α-Ni(OH)_2_ NS/CC, after 36,000 s chronoamperometric (CA) testing, SL α-Ni(OH)_2_ NS/CC achieved 131 times time enhancement (Figure 2i,j).

**Figure 3:**
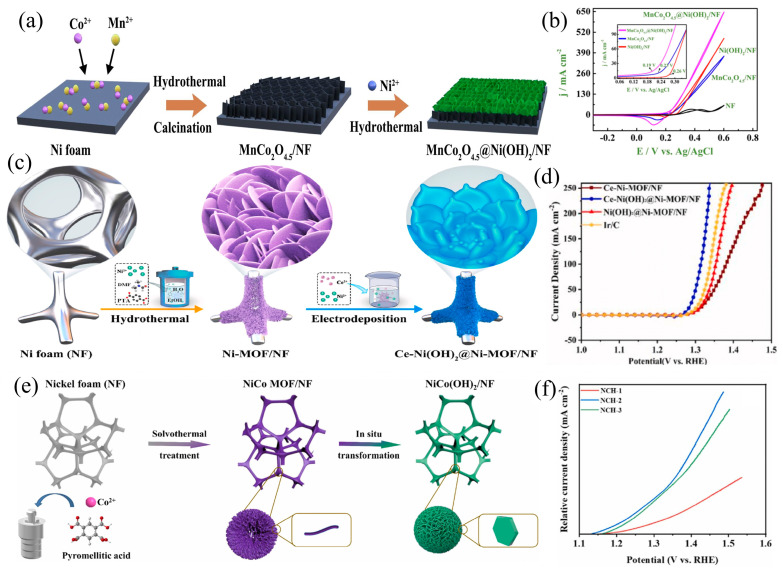
(**a**) Scheme of the synthesis and (**b**) CV of MnCo_2_O_4.5_@Ni(OH)_2_/NF, reprinted with permission from Ref. [53]. Copyright 2020, Elsevier. (**c**) Schematic diagram of the synthesis process of Ce-Ni(OH)_2_@Ni-MOF/NF and (**d**) LSV of different samples, reprinted with permission from Ref. [54]. Copyright 2024, Elsevier. (**e**) Illustration for the synthesis of nickel cobalt hydroxide and (**f**) LSV of nickel cobalt hydroxide, reprinted with permission from Ref. [55]. Copyright 2023, Elsevier.

In addition to the singular nanosheet structure, hierarchical nanosheet array structures are highlighted. Sha et al. [53] employed a continuous process involving hydrothermal treatment, annealing, and additional hydrothermal steps (denoted as MnCo_2_O_4.5_@Ni(OH)_2_/NF) to fabricate a distinctive triple-layered heterostructure (Figure 3a). In this setup, MnCo_2_O_4.5_ intricately weave together, forming strata that extend from the lower section of the NF substrate to the upper layer comprising Ni(OH)_2_ nanosheets. The plethora of active sites offered by this distinctive multilayered porous structure, coupled with its facilitation of gas release from the surface, markedly augments the activity and stability of the UOR. In the assessment of catalytic activity between MnCo_2_O_4.5_@Ni(OH)_2_/NF, MnCo_2_O_4.5_/NF, and Ni(OH)_2_/NF, the focus is directed towards elucidating the unique layered nanostructure and synergistic effects stemming from the interplay of MnCo_2_O_4.5_ and Ni(OH)_2_. Among them, the Ni(OH)_2_/NF electrode displays an increased level of current density at 650 mA cm^−2^, along with a reduced onset potential of 0.19 V vs. Ag/AgCl and remarkable robustness (Figure 3b). These findings offer valuable perspectives that contribute to the understanding of potential electrocatalytic applications. In addition to nanosheet-shaped materials, other morphology materials have been the subject of investigation as well. Cheng et al. [54] employed a facile approach to fabricate ultrathin nanosheets of Ce-Ni(OH)_2_@Ni-MOF (metal-organic frameworks) for both the OER and UOR, utilizing Ni-MOF as the precursor. Initially, they achieved the in-situ growth of nano-flower structures composed of ultrathin nanosheets of Ni-MOF on a conductive NF, demonstrating a remarkable combination of high specific surface area and numerous active sites (Figure 3c). Subsequent electro-deposition led to the successful synthesis of a composite material comprising Ni(OH)_2_ nanoparticles and Ni-MOF nanosheets, with the incorporation of Ce doping. This composite effectively preserved the pristine structure of Ni-MOF. Notably, Ce doping played a pivotal role in finely tuning the electronic structure of the active centers, facilitating efficient charge redistribution. Consequently, the resultant exhibited outstanding UOR activity, achieving 10 mA cm^−2^ at a modest potential of 1.28 V (vs. RHE, reversible hydrogen electrode) (Figure 3d). Xiang et al. [55], utilizing a nickel-based MOF as a sacrificial template, synthesized hierarchical microspheres characterized by a hexagonal NiCo(OH)_2_ nanosheet structure (Figure 3e). This intricate architecture exhibits an abundance of Ni^3+^ species and surface carboxyl groups. Attributed to its distinctive structure and the presence of diverse Ni^3+^ species and surface carboxyl groups, the synthesized catalyst exhibits outstanding UOR activity (Figure 3f).

**Figure 4:**
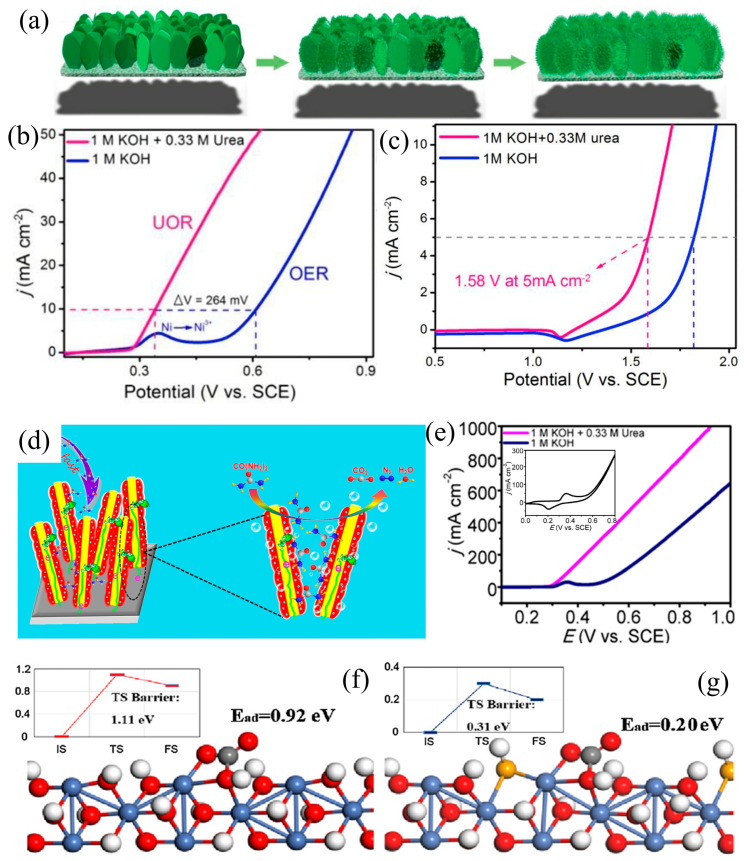
(**a**) Scheme for the synthesis and (**b**) LSV of Ni(OH)_2_ NS@NW/NF, and (**c**) OER and UOR measurements of Ni(OH)_2_ NS@NW/NF, reprinted with permission from Ref. [56]. Copyright 2018, Elsevier. (**d**) Schematic diagram of Se-Ni(OH)_2_@NiSe/NF, (**e**) LSV curves of Se-Ni(OH)_2_@NiSe/NF, and (**f**,**g**) schematic of CO_2_ adsorption on the pristine and Se-Ni(OH)_2_ (110) surface, reprinted with permission from Ref. [61]. Copyright 2017, Elsevier.

An effective method for surface engineering was introduced by Yue et al. [56], where arrays of layered Ni(OH)_2_ nanosheet@nanowire on NF (NS@NW/NF) were constructed as the anode material for urea electrolysis (Figure 4a). The developed catalyst, featuring a distinctive NS@NW architecture, demonstrated remarkable performance, requiring only 0.34 V (vs. SCE, saturated calomel electrode) potential to drive 10 mA cm^−2^ in 1.0 M KOH and 0.33 M urea (Figure 4b). This performance surpassed that of a pure nanosheet array structure. Building upon this, a dual-electrode electrolysis system was devised, incorporating a cathode composed of a nanowire array of cobalt phosphide (Co_2_P NW/NF) and using Ni(OH)_2_ NS@NW/NF as the anode for the overall urea electrolysis. At a low voltage of 1.58 V, operational efficiency is exhibited by the system, enabling the generation of 5 mA cm^−2^ containing 1.0 M KOH and 0.33 M urea. (Figure 4c). Importantly, the system demonstrated robust durability, maintaining catalytic activity for extended periods exceeding several tens of hours. The exceptional efficacy of the material can be linked to multiple contributing factors: (i) the Ni(OH)_2_ nanosheet array and NF interact closely, guaranteeing strong mechanical adhesion, effective electrical connectivity, and efficient electron transfer [57,58]; (ii) the existence of large pores in NF and the multilayered structure of Ni(OH)_2_ NS@NW arrays ensure an abundant space that facilitates the enhanced penetration of the electrolyte into numerous exposed active sites [59,60]; and (iii) the need for the utilization of poorly conductive polymer binders, such as Nafion or polytetrafluoroethylene, to encapsulate the active sites is eliminated by the streamlined synthesis process. Vertically oriented NiSe nanowires with a Se-Ni(OH)_2_ shell layer on NF were reported by Tang et al. [61] and utilized as a UOR electrocatalyst (Figure 4d,e). Numerous nanopores are formed by interconnected nanowires, as revealed by the experimental results. This structure exposes additional active sites and promotes the rapid diffusion of electrolytes and reactants to these sites. Theoretical calculations indicate that the successful establishment of high-quality transport pathways is achieved through the vertical and distant arrangement of high-porosity nanowires. High conductivity is demonstrated by the NiSe core, aiding in swift electron transfer. Abundant active catalytic sites are provided by the Se-Ni(OH)_2_ shell, with adsorption and desorption energies lower than those in Ni(OH)_2_. This contributes to the enhancement of reaction kinetics (Figure 4f,g).

Various structures of Ni(OH)_2_, such as nanosheets [62], nanoarrays [63], and nanorods [64], have been developed. Overall, morphology engineering is an important method for regulating the performance of Ni(OH)_2,_ and it has achieved significant results. By carefully designing the morphology and structure of catalysts, it is possible to effectively increase their surface area, expose active sites, and provide continuous and efficient mass transfer channels, thereby enhancing catalytic activity. In this section, we have listed some examples of morphology design and summarized their performance in the UOR (Table 1). Although morphology design offers an important way of optimizing the activity of Ni(OH)_2_ catalysts, it also presents some challenges. Firstly, complex morphology design may increase the difficulty and cost of synthesis, limiting its engineering applications. Secondly, different morphological structures may correspond to different catalytic activities and stabilities, requiring systematic optimization and comparison. Therefore, merely modifying the morphology of Ni(OH)_2_ is inadequate for designing satisfactory UOR catalysts. It is imperative to comprehensively consider catalytic performance, synthesis feasibility, and practical requirements of engineering applications. Alternative design strategies, such as doping and heterostructures, will be discussed in subsequent sections.

### 3.2. Element Doping

**Figure 5:**
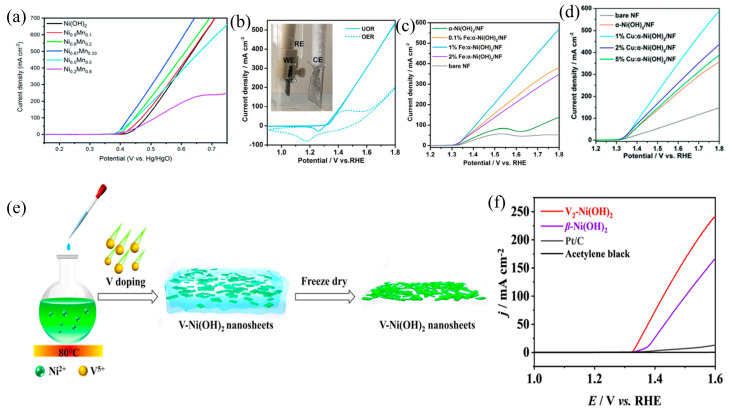
(**a**) LSV curves of different catalysts, reprinted with permission from Ref. [68]. Copyright 2022, The Royal Society of Chemistry. (**b**) OER and UOR curves for 1%Fe-Ni(OH)_2_, (**c**) LSV curves of α-Ni(OH)_2_ at different Fe doping concentrations, reprinted with permission from Ref. [63]. Copyright 2018, Wiley-VCH. (**d**) LSV curves of Cu-incorporated α-Ni(OH)_2_/NF, reprinted with permission from Ref. [71]. Copyright 2019, The Royal Society of Chemistry. (**e**) Scheme of the V_2_-Ni(OH)_2_ and (**f**) LSV curves of V_2_-Ni(OH)_2_, reprinted with permission from Ref. [72]. Copyright 2022, Elsevier.

The integration of trace elements into the catalyst elevates catalytic performance in UOR through nuanced adjustments in the catalyst’s electronic structure, optimization of interactions with active sites, and facilitation of oxidation reactions. This underscores the versatility of doping elements in the precision engineering of catalyst properties [65,66]. Widely attracting attention from researchers in various doping agents is manganese (Mn) due to its diverse oxidation states, rich redox properties, and excellent electrochemical performance. As a dopant, Mn has the capability to lower the onset potential for the conversion of Ni^2+^ to NiOOH, thereby enhancing the catalytic activity for UOR. Ni^2+^ oxidation is promoted by the hybridization of Ni 3d orbitals and O 2p orbitals facilitated by the doping of Mn, as indicated by DFT calculations [67]. Yang et al. [68] successfully synthesized Ni_0.2_Mn_0.8_ layered double hydroxides (LDHs) through electrodeposition. The facilitation of the transition from Ni^2+^ to Ni^3+^ in the electrolyte is attributed to the incorporation of Mn through doping, thereby promoting the reversibility of the Ni(OH)_2_/NiOOH redox couple and inducing an increase in Ni-O bond length and structural disorder of NiOOH under UOR potentials. This alteration allows for the generation of electrochemically active NiOOH at lower applied voltages, consequently enhancing UOR performance (Figure 5a). In previous work, it has been demonstrated that Fe effectively promotes the formation of high-valence Ni, which possesses more active electrocatalytic properties [69,70]. Consequently, catalysts based on NiFe compositions have been extensively studied for the OER. Similarly, the UOR also requires an electrochemical pre-oxidation process to generate catalytically active high-valence nickel species. Xie et al. [63] employed soluble Ni/Fe salts and urea as precursor materials to fabricate Fe-containing hierarchical α-Ni(OH)_2_ nanosheet arrays. Results from Figure 5b demonstrate that compared to the OER current density, the Fe-α(OH)_2_/NF catalyst exhibits a higher UOR current density, suggesting its enhanced efficiency in catalyzing urea oxidation. Both OER and UOR mechanisms utilize trace amounts of iron dopants to promote the formation of high-valence nickel species, thereby facilitating the reaction kinetics. However, due to urea’s nature as a more readily oxidizable organic molecule, it requires a lower oxidation potential. Consequently, Fe-α(OH)_2_/NF displays a higher UOR current density. Furthermore, the author also investigated the impact of varying Fe doping concentrations on the catalyst’s performance (Figure 5c). This exploration stems from the introduction of judicious amounts of dopants into the crystal lattice of nickel hydroxide, which induces lattice strain in the catalyst. Consequently, this strain modulates the catalyst’s electronic structure, surface characteristics, and active sites, thereby enhancing its efficacy in urea oxidation reactions. Nevertheless, the ramifications of lattice strain are twofold; while it can be advantageous, excessive or inappropriate strain induced by excessive doping can lead to catalyst instability and a subsequent decline in catalytic activity. Hence, optimizing the doping concentration of these elements is imperative during the synthesis of doped materials to ascertain the optimal doping levels. In addition to employing Fe as a dopant, Xie et al. [71] also developed a hierarchically copper-doped α-Ni(OH)_2_ nanoarray catalyst. Copper, employed as a dopant, imparts a distinctive layered sheet-on-line structure to the catalyst, thereby endowing it with abundant active sites for electrooxidation reactions. Simultaneously, the low-level doping of copper facilitates electron transfer, resulting in an outstanding electrocatalytic UOR activity (Figure 5d). The synthesized V-Ni(OH)_2_ through a co-precipitation method exhibits efficient electrocatalytic activity for the UOR, as reported by Cao et al. [72] (Figure 5e). The introduction of vanadium has been instrumental in effecting the conversion of inert β-Ni(OH)_2_ to the catalytically active α-Ni(OH)_2_, concomitantly facilitating the modulation of Ni’s electronic structure, promoting the generation of high-valence Ni^3+^ at low overpotentials. This enhancement elevated the electrocatalytic activity at various Ni^3+^ sites, accelerating the overall rate of the electrocatalytic reaction (Figure 5f).

**Figure 6:**
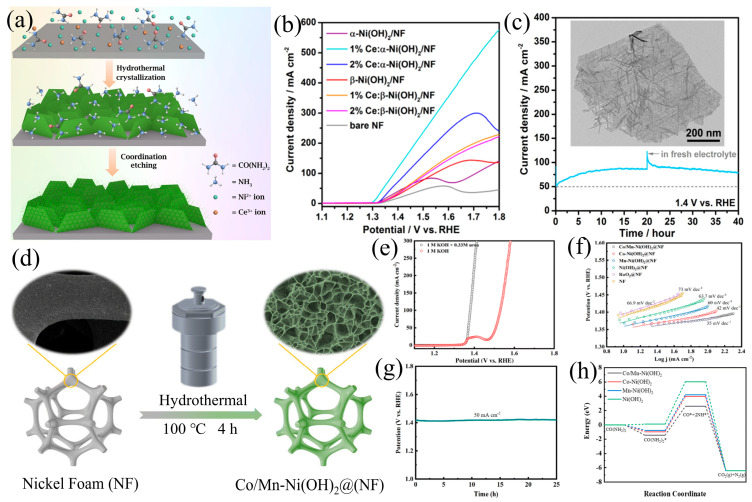
(**a**) Scheme of the formation of the hierarchical wire-on-sheet nanoarrays, (**b**) LSV for the evaluation of the UOR activity, and (**c**) CA curve of the 1% Ce:α-Ni(OH)_2_/NF and TEM after stability tests, reprinted with permission from Ref. [73]. Copyright 2019, American Chemical Society. (**d**) Scheme of the synthesis of Co/Mn-Ni(OH)_2_, (**e**,**f**) LSV, Tafel slopes and of Co/Mn-Ni(OH)_2_, and (**g**) LSV of Co/Mn–Ni(OH)_2_ before and after the 25 h stability test. (**h**) The energetic pathway comparison of the UOR, reprinted with permission from Ref. [74]. Copyright 2024, The Royal Society of Chemistry.

Xie et al. [73] proposed a novel approach combining a crystallization-coordination-corrosion mechanism to construct a distinctive on-chip linear structure. Based on this method, they successfully synthesized a Ni(OH)_2_ NW array with cerium doping and a layered on-chip linear structure for UOR (Figure 6a). In-depth studies revealed that the locally doped Ni^3+^ in the α-Ni(OH)_2_ phase exhibited higher intrinsic UOR activity compared to the β phase. Furthermore, cerium doping optimized the electronic structure, significantly enhancing the overall activity. Due to its hierarchical nanostructure, superior Ce doping, and well-engineered crystal phases, the α-Ni(OH)_2_ on-chip linear nanowire array catalyst with 1% Ce doping demonstrated remarkable UOR activity. The hierarchical α-Ni(OH)_2_ array catalyst exhibited an ultra-low initial potential of only 1.29 V vs. RHE. Moreover, under the condition of 1.8 V vs. RHE, the catalyst demonstrated a substantial basic UOR activity, reaching as high as 579.5 mA cm^−2^, as illustrated in Figure 6b,c. In addition to the extensive research on doping metal atoms with Ni(OH)_2_, the incorporation of non-metal elements has also garnered considerable attention. Beyond singular element doping, the simultaneous incorporation of multiple elements can intricately modulate the electronic structure, thereby augmenting the catalytic activity for UOR. Miao et al. [74] employed a straightforward hydrothermal synthesis method to successfully fabricate a high-performance catalyst with cobalt–manganese co-doping on NF (Co/Mn-Ni(OH)_2_, Figure 6d). The experimental results indicate that owing to its unique structure and enhanced binding strength after modification, the Co/Mn-Ni(OH)_2_ can achieve a current density of 100 mA cm^−2^ and a Tafel slope of 35 mV dec^−1^ at a potential as low as 1.38 V. By a substantial margin, this performance surpasses that observed in Ni(OH)_2_ doped with single elements. After undergoing a 25-h stability test, the catalyst was subjected to LSV measurements, revealing no significant difference before and after the test, indicating its excellent performance and stability (Figure 6e–g). The structure of nanosheets, characterized by their ultra-thin and interconnected nature, contributes to the exposure of supplementary electrochemical active sites, expediting the UOR process. DFT calculations reveal that the incorporation of both Co and Mn through dual-doping significantly decreases the energy barrier associated with the adsorption of Co(NH_2_)_2_ onto the surface of the catalyst, promoting the breakdown of the Co(NH_2_)_2_ intermediate into separate Co and NH intermediates (Figure 6h). The synergistic effect contributes to an enhancement in the UOR reaction kinetics. Additionally, the nanosheet structure, characterized by its ultra-thin and interconnected nature, facilitates the exposure of more electrochemical active sites, ultimately hastening the progression of the UOR.

**Figure 7:**
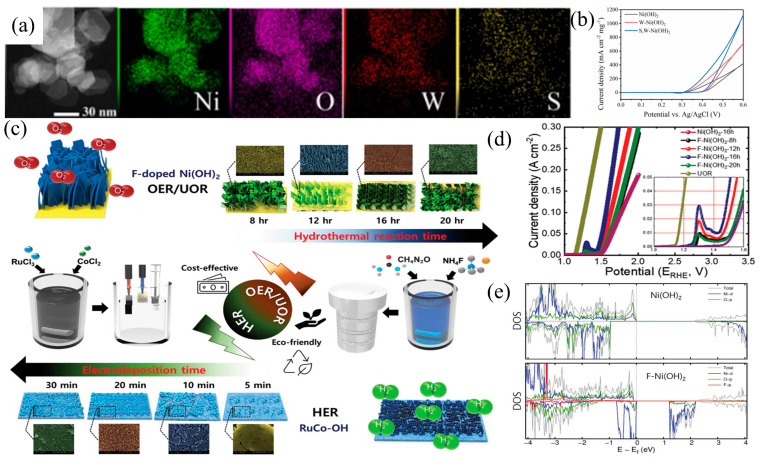
(**a**) The dark field image and the corresponding element mappings of S,W-Ni(OH)_2_ and (**b**) CV of S,W-Ni(OH)_2_, reprinted with permission from Ref. [75]. Copyright 2023, Elsevier. (**c**) Schematic diagram of the synthesis of F-Ni(OH)_2_ on NF, (**d**) LSV of the F-doped Ni(OH)_2_, and (**e**) total and projected DOS of pristine and F-doped Ni(OH)_2_ monolayers, reprinted with permission from Ref. [76]. Copyright 2022, Wiley-VCH.

Yang et al. [75] incorporated sulfur into Ni(OH)_2_, creating a catalyst with a lamellar structure and a substantial BET surface area. The sulfur doping enhances both charge transfer and urea adsorption capabilities, resulting in outstanding performance in UOR (Figure 7a,b). Patil et al. [76] employed an ecologically friendly one-step synthesis method to successfully fabricate self-supporting, fluorine-modified 2D nanosheets composed of ultrafine Ni(OH)_2_, replacing outdated high-vacuum and high-temperature processes (Figure 7c). The heterogeneous catalyst demonstrated heightened redox activity and conductivity, coupled with a substantial presence of readily accessible active sites, enhancing charge transfer in electrocatalytic reactions. DFT calculations revealed a significant increase in the density of Ni atom d-orbitals near the Fermi level, suggesting that F-doping in the Ni(OH)_2_ catalyst resulted in a finely tuned electronic surface structure and increased conductivity due to the generated defect levels. Furthermore, the introduction of oxygen vacancies into the catalytic material lattice resulted in the revelation of more active sites on the catalyst surface, thereby further influencing electrocatalytic performance. The prepared catalyst demonstrated exceptional UOR activity (Figure 7d,e).

Therefore, element doping, as a crucial means to regulate the performance of Ni(OH)_2_ catalysts, demonstrates immense potential in enhancing catalytic activity and stability. By introducing other elements into the crystal structure of Ni(OH)_2_, one can adjust the catalyst’s electronic structure, surface properties, and active sites, thereby significantly improving its performance in UOR. In this section, we have presented several examples of element doping and summarized their performance in UOR (Table 2). These studies indicate that the introduction of dopants can modulate the catalyst’s electronic structure, optimize the desorption and adsorption energies of intermediates, and reduce the onset potential for Ni^2+^ to convert into Ni^3+^, consequently enhancing the catalytic activity of UOR.

Significant progress has been made in utilizing element doping into Ni(OH)_2_; there are also several limitations, including (i) the effects of doping with different elements may vary, necessitating systematic optimization and comparison, (ii) some element doping may increase the difficulty and cost of synthesis, thereby limiting their engineering applications, and (iii) the synergistic effect between dopants and catalysts is one of the factors contributing to the enhanced performance of UOR catalysts. However, the “synergistic effects” are often used to explain without providing additional detailed evidence or elucidating the specific mechanisms underlying bimetallic synergy in most cases.

### 3.3. Defect Engineering

**Figure 8:**
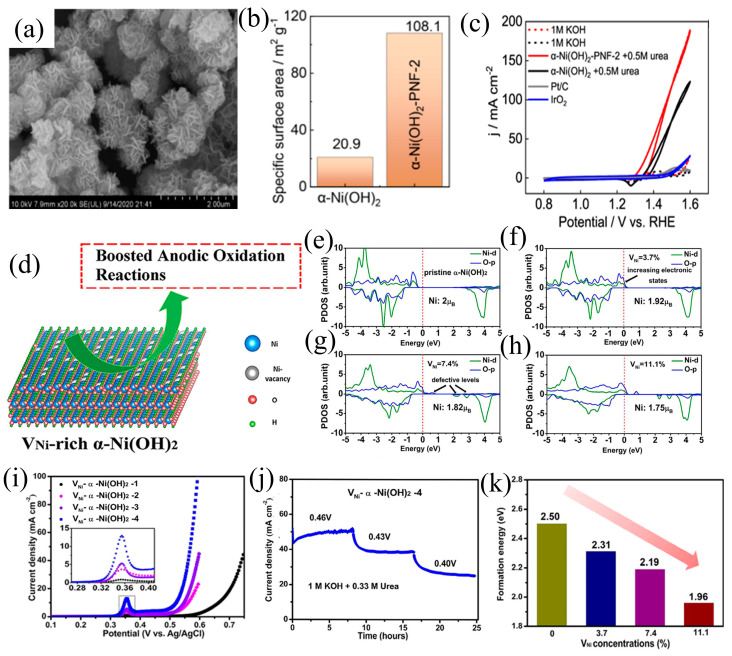
(**a**) TEM images of α-Ni(OH)_2,_ (**b**) CO_2_-TPD of two electrocatalysts, and (**c**) CV of α-Ni(OH)_2_, reprinted with permission from Ref. [81]. Copyright 2021, Elsevier. (**d**) Diagram of V_Ni_ rich α-Ni(OH)_2_, (**e**–**h**) DOS spectra of α-Ni(OH)_2_ with V_Ni_ different concentrations, and (**i**,**j**) LSV and long-term tests for V_Ni_-α-Ni(OH)_2−x_. (**k**) The calculated formation energies for NiOOH from α-Ni(OH)_2_ with different V_Ni_ concentrations, reprinted with permission from Ref. [32]. Copyright 2018, American Chemical Society.

The strategic manipulation of defect processes plays a crucial role in catalyst design, enhancing catalytic performance through tailored structural modifications [77,78,79,80]. Oxygen vacancies, as a form of point defect, represent a commonly employed strategy in the synthesis of defect-engineered catalysts within catalyst frameworks. Building upon the approach, Li et al. [81] successfully synthesized α-Ni(OH)_2_ porous nanoflowers (α-Ni(OH)_2_-PNF), where the Kirkendall effect led to the creation of nano-flower structures (Figure 8a). This specific morphology played a pivotal role in promoting the generation of abundant oxygen vacancies during the UOR, simultaneously reducing mass transport resistance and regulating the electronic structure of active centers (Figure 8b). The introduction of these oxygen vacancies effectively decreased the binding strength between CO_2_ and the catalyst, resulting in an improved diffusion performance of the electrolyte under high current density. This meticulous adjustment ultimately culminated in remarkable catalytic performance, exemplified by a current density of 100 mA cm^−2^ achieved at 1.477 V vs. RHE (Figure 8c). Rooted in this conceptual framework, the deliberate introduction of defects on the catalytic surface proves instrumental in fully exposing a greater number of active sites.

Beyond oxygen vacancies, the creation of alternative defect types further amplifies the activity in urea oxidation. He et al. [32] devised a controlled hydrolysis strategy to synthesize Ni(OH)_2_ catalysts featuring adjustable concentrations of nickel vacancies (V_Ni_) (Figure 8d). Electrochemical measurements demonstrated that an augmentation in V_Ni_ concentration expedited the reconstruction process, yielding authentic active components and thereby amplifying catalytic activity for both the OER and UOR while also conferring excellent stability upon the catalyst. DFT simulations revealed that the introduction of V_Ni_ enhances the conductivity of Ni(OH)_2_, fostering the formation of active species and expediting the electrochemical oxidation process (Figure 8e–j). The heightened V_Ni_ concentration was observed to diminish the formation energy of genuine active species during the reaction (Figure 8k). This work underscores the synergy of defect engineering strategies, atomic-level spectroscopic characterization, and theoretical calculations, offering profound insights into the intricate structure–activity dynamics of electrocatalysts. Furthermore, the introduction of outer elements may instigate the formation of vacancies and defects. Qin et al. [82] synthesized Ni(OH)_2_ enriched with oxygen vacancies and doped with vanadium (O_vac_-V-Ni(OH)_2_). The integration of theoretical calculations with experimental findings elucidates that V doping not only exposes additional intrinsic active sites for the UOR but also modulates the electronic state of NiOOH, thereby mitigating the chemical adsorption energy between intermediates and the catalyst surface.

**Figure 9:**
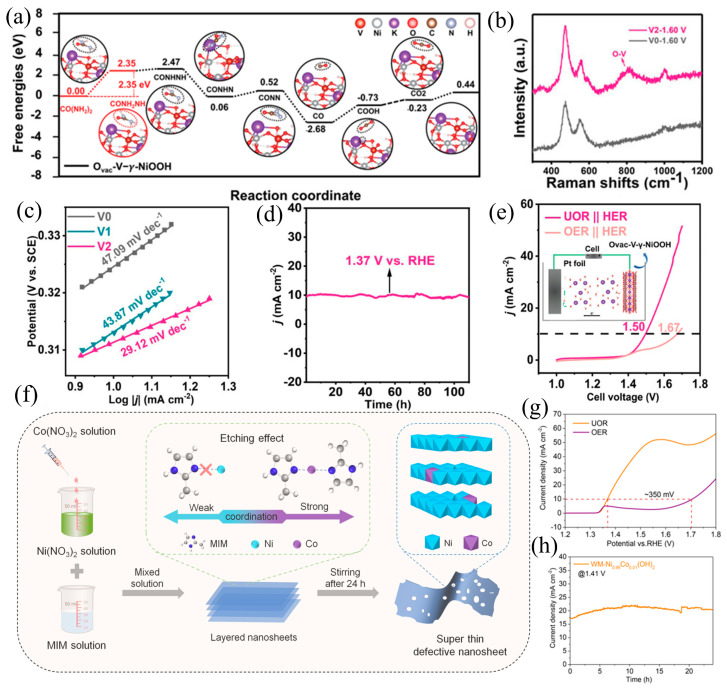
(**a**) The Gibbs free energy profiles of the proposed UOR pathway at NiOOH, (**b**) Raman spectra of V_0_ and V_2_, (**c**) Tafel slopes for the V_0_, V_1,_ and V_2_, (**d**) i–t curve of V_2_ sample, and (**e**) LSV curves for urea electrolysis and water electrolysis, reprinted with permission from Ref. [82]. Copyright 2023, Wiley-VCH. (**f**) Synthesis of defective NiCo(OH)_2_, (**g**) LSV of NiCo(OH)_2_, and (**h**) CA of NiCo(OH)_2_, reprinted with permission from Ref. [83]. Copyright 2023, Wiley-VCH.

In-situ Raman spectroscopy unveiled structural disorder and diminished crystallinity resulting from vanadium doping. Throughout the electrochemical reconstruction process, alterations in the Raman peak intensity ratio indicated the formation of γ-NiOOH (Figure 9a,b). Utilizing the synergistic effects of doping and vacancies, the NiOOH catalyst demonstrated a reduced reaction barrier (decreasing from 3.48 to 2.35 eV), showcasing enhanced reaction kinetics and persistent UOR activity. The stability of V_2_ in UOR is further underscored by its significantly lower Tafel slope of 29.12 mV dec^−1^, in contrast to V_0_ (47.09 mV dec^−1^) and V_1_ (43.87 mV dec^−1^) in a 1.0 M KOH and 0.33 M urea (Figure 9c,d). In addition, the authors applied the prepared catalyst to the electrolysis of urea and water (with Pt as the cathode) in practical applications. When the current density of the urea electrolysis system reached 10 mA cm^−2^, the cell voltage was only 1.5 V, significantly lower than the 1.67 V required by traditional water electrolysis systems, thereby saving energy in the electrolysis of water for H_2_ production (Figure 9e). This work underscores that dual-center engineering involving heteroatom doping and vacancies serves to bridge the gap between selective design and catalyst surface science. Liu et al. [83] employed a dual solvent system, combining water and methanol for the first time. They utilized minute quantities of cobalt doping to initiate defect engineering in α-Ni(OH)_2_, resulting in the synthesis of co-doped α-Ni(OH)_2_ (WM-Ni_1−x_Co_x_(OH)_2_, Figure 9f). The comprehensive physical characterization of WMNi_0.99_Co_0.01_(OH)_2_ revealed a pronounced abundance of defects compared to the original catalyst. The strategic combination of co-doping and defects efficiently fine-tuned the electronic structure, facilitating the formation of Ni^3+^, which is a crucial aspect in mitigating overpotential during the UOR. Subtle alterations in surface chemical states induced a notable reduction in the bandgap, accompanied by a significant increase in the specific surface area from 68 to 172.3 m^2^ g^−1^. As a result, WM-Ni_0.99_Co_0.01_(OH)_2_ demonstrated a substantial reduction of nearly 110 mV (1.47 V vs. RHE) at a current density of 10 mA cm^−2^ and exhibited enduring stability during 24 h of continuous operation (Figure 9g,h). Precision control over the location and concentration of defects enables the tailored design of UOR catalysts with strengthened catalytic activity.

This section provides several examples of successful applications of defect engineering in Ni(OH)_2_ catalysts (Table 3). Defect engineering, achieved through the introduction of lattice defects or control of crystal structure, allows for the adjustment of surface-active sites and electron transfer rates of the catalyst, thereby enhancing its activity. However, the presence of high-density defects may lead to catalyst instability and deactivation. Furthermore, the correlation between different types of defects and catalytic activity requires further investigation. Hence, more in-depth research is necessary to understand the impact mechanisms of various defect types on catalyst performance, facilitating more effective catalyst design and optimization.

### 3.4. Heterostructure Construction

**Figure 10:**
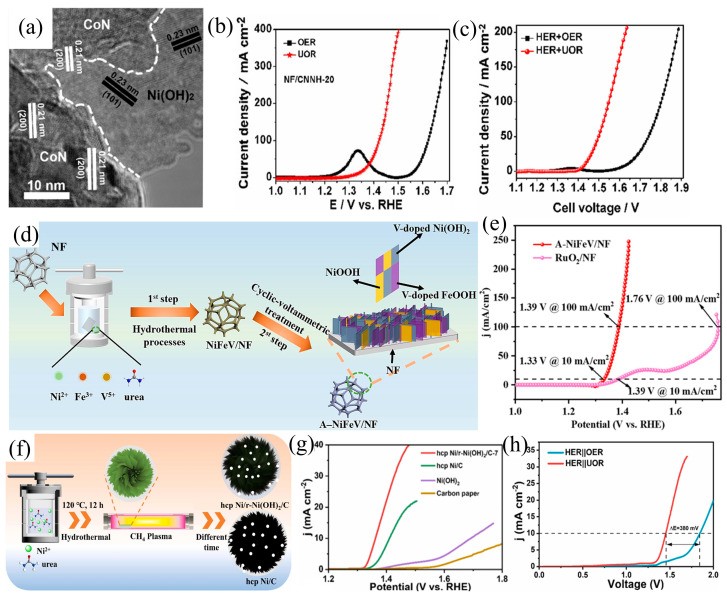
(**a**) HRTEM image of CoN/Ni(OH)_2_, (**b**) LSV of CoN/Ni(OH)_2_, and (**c**) LSV of CoN/Ni(OH)_2_ for overall water electrolysis and urea electrolysis, reprinted with permission from Ref. [89]. Copyright 2020, Elsevier. (**d**) Scheme of the NiFeV/NF, (**e**) LSV of NiFeV/NF, reprinted with permission from Ref. [91]. Copyright 2024, Elsevier. (**f**) Schematic illustration of the formation of Ni/r-Ni(OH)_2_, (**g**) LSV of different catalysts, and (**h**) polarization curves of Ni/r-Ni(OH)_2_/C electrocatalysts in urea and water electrolysis, reprinted with permission from Ref. [92]. Copyright 2023, Elsevier.

Constructing Schottky heterojunctions serves as a powerful method to tailor urea electrocatalysts, harnessing the intrinsic electric field initiated by heterojunction interface curvature to facilitate charge redistribution and self-driven charge transfer, thereby enhancing urea oxidation by bolstering adsorption capacity for -NH_3_ and -C=O groups and yielding superior mechanical and physical properties compared to traditional composite materials [84,85,86,87]. However, achieving efficient multi-component electrocatalysts remains a challenge due to the sluggish dynamics of charge transfer at the interface. Previous studies have suggested that the polarization of crystal planes may facilitate interface charge transfer [88]. Therefore, Cheng et al. [89] utilized a hydrothermal–nitridation–electrodeposition process to fabricate a dual-functional electrocatalyst (referred to as NF/CNNH) on NF, incorporating Ni(OH)_2_ and CoN components. The formed heterostructure of CoN/Ni(OH)_2_ consists of CoN (111) and Ni(OH)_2_ (001), with a reduced lattice mismatch of only 2.8%, indicating minimal deformation in the CoN/Ni(OH)_2_ heterostructure (Figure 10a). The polar plane of CoN (111) facilitates interface charge transfer, thereby promoting the electrocatalytic process. This catalyst demonstrates exceptional UOR activity, achieving a potential of 1.39 V vs. RHE at a current density of 50 mA cm^−2^, with a Tafel slope of 64 mV dec^−1^ (Figure 10b). Assembling CoN/Ni(OH)_2_ as both the anode and cathode form a dual-electrode urea electrolyzer, requiring a cell voltage of less than 1.43 V to drive a current density of 10 mA cm^−2^, demonstrating remarkable stability (Figure 10c). In addition to the design strategy involving vacancies and multiple components combined with heterogeneous structures, catalysts that integrate atomic doping with heterogeneous structures are also crucial for enhancing electrochemical performance. The doping of foreign metals and the interaction at the interface enhance the electronic conductivity of the heterogeneous structure, promoting the dynamics of charge transfer at the interface and consequently improving electrochemical performance. Vanadium exhibits low electronegativity and possesses a surplus of vacant d orbitals. This unique combination significantly influences the electronic structure of the target catalyst, thereby exerting a pronounced effect on its catalytic performance [90]. Yang et al. [91] conducted a synthesis of a heterogeneous electrocatalyst, V-doped Ni(OH)_2_/FeOOH, utilizing a dual-phase approach that encompasses hydrothermal deposition followed by electrochemical activation, named A-NiFeV/NF (“A” indicating “activated”, Figure 10d). In this structure, atomic doping and heterogeneous interfaces coexist. Electrochemical evaluations unveil that the customized A-NiFeV/NF electrocatalyst exhibits reduced overpotentials under 10 and 100 mA cm^−2^, registering 1.33 and 1.39 V vs. RHE (Figure 10e). Gao et al. [92] harnessed a hybrid approach involving hydrothermal reduction and in situ topological reduction, culminating in the proficient fabrication of nanosheets featuring an ultra-thin carbon layer and hexagonal close packing (hcp) Ni/r-Ni(OH)_2_ with a notable abundance of oxygen defects (Figure 10f). The catalyst showcases intricate interface synergies that promote efficient electron transfer between transition metal hydroxides and their corresponding transition metals, facilitating the modulation of urea adsorption. Demonstrating exceptional efficiency, the electrocatalyst based on hcp Ni/r-Ni(OH)_2_/C exhibits a potential of 1.36 V at 10 mA cm^−2^ during UOR and displays commendable activity, achieving a potential of 1.45 V at 10 mA cm^−2^ in urea electrolysis (Figure 10g,h). The authors attribute the high performance of this catalyst to several factors, such as (i) Optimizing the surface electronic structure of Ni involves the redistribution of charges at the interface between hcp and r-Ni(OH)_2_ and Ni, promoting the adsorption of urea and H_2_O, enhancing the intrinsic activity of UOR; (ii) The nanosheet structure exposes numerous active sites, further improving the UOR activity; (iii) Mesoporous structure enhances mass transfer capability, thereby increasing the overall activity. The energy level difference at the heterostructure interface induces a self-driven electron transfer effect, facilitating rapid electron transfer and enhancing reaction kinetics. Simultaneously, the interaction between heterostructures, influenced by their unique 3D morphology, assists in modulating the electronic structure and exposes additional active centers, further synergistically optimizing catalytic activity.

Heterostructures, as a classical strategy for modulating catalyst performance, exhibit diversity and significant potential. They are not merely a simple integration of different components; rather, their enhanced UOR activity stems from the augmented charge transfer energy at the hetero-interface. In this section, we presented several successful cases of leveraging heterostructures (Table 4). These findings underscore the effective utilization of heterostructures in harnessing the distinct advantages of various materials, thereby notably enhancing the performance and stability of Ni(OH)_2_ catalysts. Despite the considerable promise of heterostructures in catalyst design, the comprehension understanding of interface structures remains incomplete. DFT calculations have been employed to elucidate the catalytic mechanisms of heterostructure catalysts, but theoretical computations still fall short of fully replicating the authentic operational conditions of catalyst systems. Consequently, further in-depth studies of interfaces are warranted to comprehensively grasp the impact of heterostructures on catalyst performance.

## 4. Challenges and Outlook

**Figure 11:**
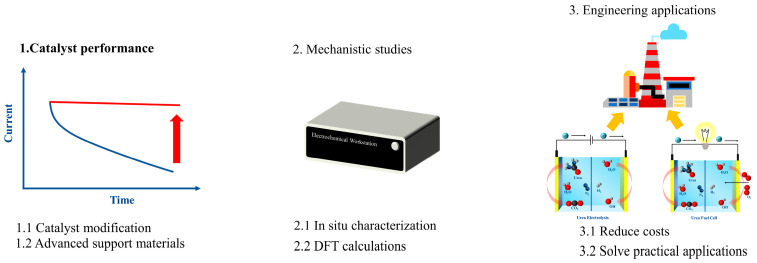
Future directions for urea oxidation electrocatalysts.

The utilization of Ni(OH)_2_ in UOR has undergone extensive investigation, yielding notable advancements. However, a myriad of challenges persists. In laboratory settings, addressing the long-term stability of catalysts and elucidating uncertainties in reaction mechanisms are imperative. Furthermore, in industrial applications, overcoming the high production costs of catalysts and the intricacies associated with synthesis processes remains paramount. Beyond these focal points, additional considerations encompass the activity and selectivity of catalysts alongside their activity across varying conditions. Addressing these challenges mandates interdisciplinary collaboration and in-depth experimental study to propel the advancement of this field. In future research, emphasis is recommended on the following aspects (Figure 11):

(1) Further refinement of catalysts: enhancing the performance of Ni(OH)_2_ catalysts, particularly concerning long-term stability in UOR, through rational design and synthesis.
(2) Mechanistic studies and surface science: undertaking more detailed characterization studies and theoretical investigations to comprehend the reaction mechanisms of nickel hydroxide catalysts in urea oxidation, thereby revealing active sites and reaction pathways for more informed design.
(3) Practical applications: researchers are urged to transcend laboratory confines, engage in collaboration with industrial partners, and translate laboratory achievements into practical applications. This necessitates addressing engineering challenges and evaluating the practical effectiveness of Ni(OH)_2_ in various applications, including H_2_ production, wastewater treatment, and fuel cells.

## 5. Conclusions

Urea, ubiquitously present as both a biological waste and a versatile commodity, holds substantial promise as an energy carrier for electricity generation in fuel cells or hydrogen production in electrochemical reactors. The imperative for sustainable energy applications through urea electrooxidation necessitates the development of efficient and cost-effective catalysts. Through a comprehensive review of nickel hydroxide (Ni(OH)_2_) materials in urea oxidation, this study provides insights into the progress and challenges in this field. From the literature survey, the following conclusions can be drawn. Firstly, nickel hydroxide demonstrates commendable performance as a catalyst for urea oxidation, manifesting significantly improved activity and stability. This establishes the groundwork for the development of more efficient, economical, and sustainable urea oxidation processes. Secondly, the critical role of material design, encompassing catalyst morphology, structural design, surface active sites, and surface energy regulation, is acknowledged. This recognition provides an array of directions for future research to optimize the performance of nickel hydroxide. This paper also puts forth certain perspectives on the challenges encountered by nickel hydroxide in urea oxidation, with the aim of offering insights for future research in this area. In conclusion, substantial progress has been achieved in the study of nickel hydroxide in urea oxidation, yet unknowns and potential opportunities persist. Through continuous research and innovation, confidence is placed in realizing more sustainable and efficient urea oxidation processes in the future, thereby contributing to the development of new energy sources.

## Figures and Tables

**Table 1:** Performance of morphology-engineered catalysts in urea electrolysis.

Catalyst	KOH and Urea	Potential/V	Current Density/mA cm^−2^	ECSA or Tafel Slope	Ref.
Ni(OH)_2_-NMs	1 M and 0.33 M	1.35 (vs. RHE)	10	231 mV dec^−1^	[51]
SL α-Ni(OH)_2_ NS/CC	1 M and 0.33 M	0.4 (Ag/AgCl)	100	130.2 mF cm^−2^	[52]
MnCo_2_O_4.5_@Ni(OH)_2_/NF	5 M and 0.33 M	0.6 (Ag/AgCl)	650	29.0 mF cm^−2^	[53]
Ce-Ni(OH)_2_@Ni-MOF/NF	1 M and 0.5 M	1.28 (vs. RHE)	10	24.65 mV dec^−1^	[54]
NiCo(OH)_2_	1 M and 0.33 M	1.368 (vs. RHE)	100	231 mV dec^−1^	[55]
Ni(OH)_2_ NS@NW/NF	1 M and 0.33 M	1.58 (vs. SCE)	5	47 mV dec^−1^	[56]
Se-Ni(OH)_2_@NiSe/NF	1 M and 0.33 M	0.366 (vs. SCE)	100	31.2 mF cm^−2^	[61]

**Table 2:** Performance of doping-engineered catalysts in urea electrolysis.

Catalysts	KOH and Urea	Potential/V	Current Density	ECSA or Tafel Slope	Ref.
Ni_0.2_Mn_0.8_ LDHs	1 M and 0.33 M	0.44 (vs. Hg/HgO)	100 mA cm^−2^	23.8 mV dec^−1^	[68]
Fe-α(OH)_2_/NF	1 M and 0.33 M	1.408 (vs. RHE)	100 mA cm^−2^	35 mV dec^−1^	[63]
Cu-α-Ni(OH)_2_/NF	1 M and 0.33 M	1.45 (vs. RHE)	100 mA cm^−2^	1.32 mF cm^−2^	[71]
V-Ni(OH)_2_	1 M and 0.33 M	1.6 (vs. RHE)	241 mA cm^−2^	32.15 mV dec^−1^	[72]
S,W-Ni(OH)_2_	1 M and 0.33 M	0.6 (Ag/AgCl)	100 mA cm^−2^	106.1 mF cm^−2^	[75]
1% Ce:α-Ni(OH)_2_/NF	1 M and 0.33 M	1.8 (vs. RHE)	579.5 mA cm^−2^	25 mV dec^−1^	[73]
Co/Mn-Ni (OH)_2_	1 M and 0.33 M	1.38 (vs. RHE)	100 mA cm^−2^	35 mV dec^−1^	[74]
F-Ni(OH)_2_	1 M and 0.33 M	1.16 (vs. RHE)	10 mA cm^−2^	29.36 mV dec^−1^	[76]

**Table 3:** Performance of defect-engineered catalysts in urea electrolysis.

Catalysts	KOH and Urea	Potential/V	Current Density	ECSA or Tafel Slope	Ref.
α-Ni(OH)_2_-PNF	1 M and 0.5 M	1.477 (vs. RHE)	100 mA cm^−2^	7.0 mF cm^−2^	[81]
V_Ni_-α-Ni(OH)_2_	1 M and 0.33 M	0.36 (vs. Ag/AgCl)	10 mA cm^−2^	29.7 mV dec^−1^	[32]
O_vac_-V-Ni(OH)_2_	1 M and 0.33 M	1.47 (vs. RHE)	100 mA cm^−2^	29.12 mV dec^−1^	[82]
WM-Ni_0.99_Co_0.01_(OH)_2_	1 M and 0.33 M	1.37 (vs. RHE)	10 mA cm^−2^	31 mV dec^−1^	[83]

**Table 4:** Performance of heterostructure-engineered catalysts in urea electrolysis.

Catalysts	KOH and Urea	Potential/V	Current Density	ECSA or Tafel Slope	Ref.
CoN/Ni(OH)_2_	1 M and 0.5 M	1.39 (vs. RHE)	50 mA cm^−2^	64 mV dec^−1^	[89]
A-NiFeV/NF	1 M and 0.33 M	1.39 (vs. RHE)	100 mA cm^−2^	34.8 mV dec^−1^	[91]
hcp Ni/r-Ni(OH)_2_	1 M and 0.33 M	1.36 (vs. RHE)	100 mA cm^−2^	52.73 mV dec^−1^	[92]

## Data Availability

Not applicable.
